# Fucoxanthin from *Laminaria japonica* Targeting PANoptosis and Ferroptosis Pathways: Insights into Its Therapeutic Potential Against Ovarian Cancer

**DOI:** 10.3390/md23030123

**Published:** 2025-03-12

**Authors:** Yaze Wang, Yiru Mao, Hui Liu, Yi Huang, Rong Xu

**Affiliations:** 1 Department of Pharmacology, School of Basic Medicine, Tongji Medical College, Huazhong University of Science and Technology, Wuhan 430030, China; yaze8263@163.com (Y.W.); u202113286@hust.edu.cn (Y.M.); liuh@hust.edu.cn (H.L.); 2 The Key Laboratory for Drug Target Researches and Pharmacodynamic Evaluation of Hubei Province, Wuhan 430030, China; 3 Biomedical Analysis Center, Army Medical University, Chongqing 400038, China

**Keywords:** fucoxanthin, ovarian cancer, programmed cell death, PANoptosis, ferroptosis, AMPK/Nrf2/HMOX1 axis

## Abstract

Ovarian cancer (OC) is a highly aggressive malignancy with a poor prognosis, necessitating novel therapeutic strategies. Fucoxanthin (FX), a marine-derived carotenoid from *Laminaria japonica*, has demonstrated promising anticancer potential. This study revealed that FX exerts multiple anticancer effects in OC by inhibiting cell proliferation, invasion, and migration, while inducing various forms of programmed cell death (PCD). FX triggered PANoptosis (apoptosis, necroptosis, and pyroptosis) and ferroptosis. FX treatment regulated key markers associated with PANoptosis, including apoptosis (Bcl-2, cleaved caspase-3), pyroptosis (GSDME), and necroptosis (RIPK3). Additionally, FX treatment modulated ferroptosis-related markers, such as SLC7A11 and GPX4, while increasing reactive oxygen species (ROS) and Fe^2+^ levels and disrupting mitochondrial function. Proteomic and molecular docking analyses identified AMP-activated protein kinase (AMPK) as a direct FX target, activating the AMPK/Nrf2/HMOX1 pathway to promote ferroptosis. In vivo, FX significantly reduced tumor growth in OC xenograft models, accompanied by enhanced ferroptosis marker expression. These findings demonstrate that FX induces ferroptosis through the AMPK/Nrf2/HMOX1 pathway and promotes PANoptosis via distinct mechanisms, highlighting its potential as a marine-derived therapeutic agent for OC.

## 1. Introduction

Ovarian cancer (OC) is one of the most lethal gynecologic malignancies, ranking as the leading cause of death among female reproductive system cancers [1]. Despite advances in treatment, including platinum-based chemotherapy (e.g., carboplatin) combined with paclitaxel [2] and targeted therapies such as bevacizumab and PARP inhibitors (olaparib, niraparib), many patients are diagnosed at advanced stages, limiting therapeutic efficacy and leading to high recurrence rates [3]. Moreover, ovarian cancer is characterized by strong drug resistance, with many patients developing resistance to platinum-based chemotherapy and targeted therapies over time, further compromising treatment outcomes and contributing to poor prognosis [4]. The urgent need for novel therapeutic strategies has driven increasing interest in discovering new treatment approaches based on emerging mechanisms for inhibiting OC.

PANoptosis, a novel type of programmed cell death (PCD), is essential in cancer pathogenesis and therapy [5,6]. While apoptosis, necroptosis, and pyroptosis have traditionally been recognized as separate signaling pathways, recent evidence suggests their interconnection at various levels, leading to the formation of the PANoptosome complex and the subsequent activation of PANoptosis [7]. Similarly, ferroptosis, a distinct iron-dependent PCD mechanism characterized by lipid peroxidation [8], has emerged as a promising therapeutic target in OC, given the cancer’s heightened iron metabolism [9,10]. In OC cells, both iron uptake and retention increase [11], making them particularly susceptible to erastin-induced ferroptosis [12]. Recent research has demonstrated the feasibility and therapeutic potential of explicitly targeting PANoptosis and ferroptosis to impede cancer growth. As reported in the literature, chrysoeriol, a natural flavonoid, induces PANoptosis and ferroptosis in melanoma cells [13]. Currently, a new perspective is emerging regarding how PANoptosis and ferroptosis influence ovarian cancer development.

Marine-derived compounds have emerged as a valuable source of therapeutics for a broad range of diseases due to their structural diversity and potent bioactivities [14]. Many marine-derived secondary metabolites exhibit cytotoxic, pro-apoptotic, and anti-metastatic properties, making them promising candidates for cancer treatment [15]. Notably, marine carotenoids have gained increasing attention for their antioxidant, anti-inflammatory, and anticancer effects [16]. Fucoxanthin (FX) is a brown-colored xanthophyll carotenoid predominantly found in *Laminaria japonica*, *Undaria pinnatifida*, and some diatoms [17]. It has garnered significant attention due to its diverse biological activities [18], including the inhibition of pre-adipocyte differentiation [19], prevention of mutagenesis [20], anti-inflammatory effects, and antioxidant properties [21]. In addition, FX has also been investigated for its potential in developing safe medicines and nutraceuticals for lifestyle-related diseases such as hypertension, stroke, type 2 diabetes, and osteoporosis [22]. Furthermore, FX has demonstrated anticancer effects across various cancer types, including ovarian, lung, leukemia, cutaneous, colon, liver, prostate, and breast cancers [23,24,25,26,27]. Our previous investigations revealed that FX inhibits the metastasis and drug sensitivity of lung cancer [28,29]. Recent studies have specifically explored its efficacy in ovarian cancer cell models. For example, FX has been shown to enhance therapeutic sensitivity in multidrug-resistant ovarian cancer cells through multiple mechanisms, including promoting apoptosis, inhibiting the expression of drug-resistant proteins, regulating metabolic enzymes, and inhibiting the epithelial–mesenchymal transition (EMT) pathway [30,31,32,33]. These studies suggest that fucoxanthin, FX, can exert conventional apoptosis-inducing and drug-resistant protein inhibition in ovarian cancer cells. Recent investigations have elucidated fucoxanthin’s inhibition of tumor growth in OC by deactivating the STAT3/c-Myc pathway [34]. However, the potential of FX-induced PANoptosis and ferroptosis in ovarian cancer remains unclear.

The objective of this study is to evaluate the therapeutic potential of FX in OC and elucidate its underlying mechanisms. We provide evidence that two specific forms of PCD, PANoptosis and ferroptosis, are important in FX-induced OC cell death, highlighting its potential as a marine-derived anticancer bioactive compound.

## 2. Results

### 2.1. Fucoxanthin Suppresses the Proliferation, Migration, and Invasion of Ovarian Cancer Cells

**Figure 1:**
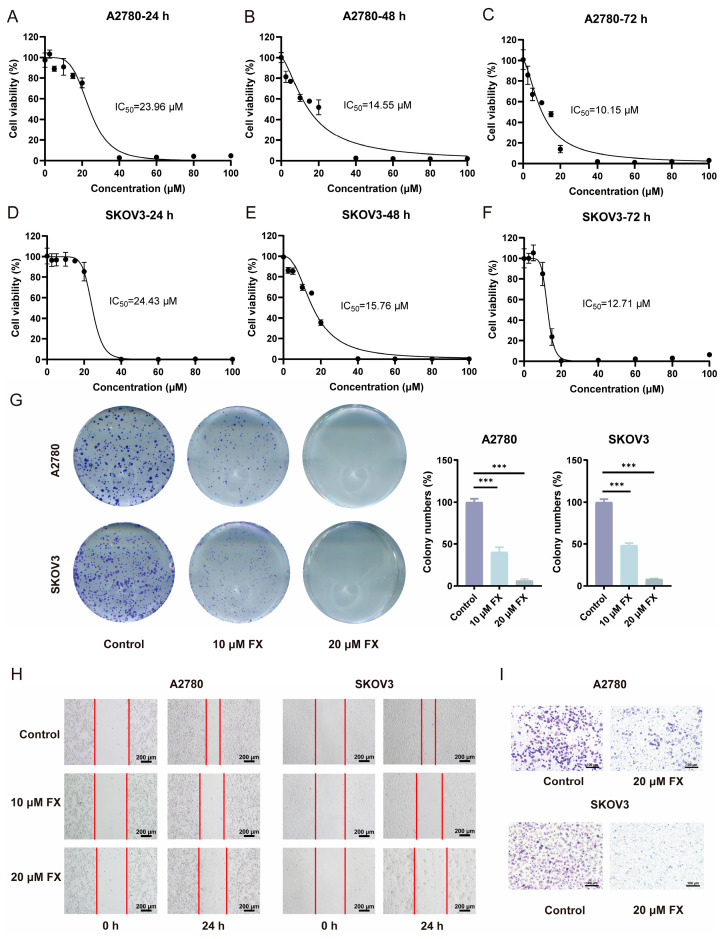
FX inhibits the proliferation and metastasis of A2780 and SKOV3 cells. (**A**–**F**) FX caused a decrease in the viability of A2780 (**A**–**C**) and SKOV3 (**D**–**F**) cells. Varied concentrations of FX (0, 2.5, 5, 10, 15, 20, 40, 60, 80, and 100 μM) were administered to the cells for 24, 48, and 72 h. (**G**) FX suppressed the proliferation of A2780 and SKOV3 cellular colonies. (**H**) The migration abilities of A2780 and SKOV3 cells with FX were assessed by wound healing assays. (**I**) The invasion abilities of A2780 and SKOV3 cells with FX were evaluated by transwell assays. The results are shown as the average value plus or minus the standard error of the mean (SEM), *n* = 3. *** *p* < 0.001, when compared to the control group.

To assess the effects of FX on the proliferation of ovarian cell lines, we exposed ovarian cancer cells A2780 and SKOV3 to varying concentrations of FX for durations of 24, 48, and 72 h, subsequently evaluating cell viability using the MTT assay. Consequently, FX treatment exhibited a time- and dose-dependent reduction in the viability of ovarian cancer cells. The IC50 values of FX following 24 h of treatment were determined to be 23.96 and 24.43 μM in A2780 and SKOV3 cells, respectively (Figure 1A,D). The IC50 values of FX following 48 h of treatment were determined to be 14.55 and 15.76 μM in A2780 and SKOV3 cells, respectively (Figure 1B,E). The IC50 values of FX following 72 h of treatment were determined to be 10.15 and 12.71 μM in A2780 and SKOV3 cells, respectively (Figure 1C,F). These findings underscore the cytotoxic potential of FX in OC. Fucoxanthin exhibited significantly reduced cytotoxicity in 293T cells (human embryonic kidney epithelial cells) compared to ovarian cancer cell lines, with parallel safety profiles observed in L02 hepatocytes (Figure S1). Furthermore, A2780 and SKOV3 cells were treated with 10 and 20 μM FX for 48 h. Two weeks later, a colony formation assay was conducted. Treatment with FX significantly decreased the proliferation of A2780 and SKOV3 cells, as shown by the decreased colony formation (Figure 1G). Transwell and wound healing assays demonstrated that FX effectively reduced the migratory and invasive potential of OC cell lines, highlighting its suppressive effects on tumor progression (Figure 1H,I). Our findings elucidate FX’s suppressive effects on tumor cell growth, migration, and invasion in A2780 and SKOV3 cells.

### 2.2. Proteomics-Based Study of the Mechanism of FX-Induced Programmed Cell Death in Ovarian Cancer Cells

**Figure 2:**
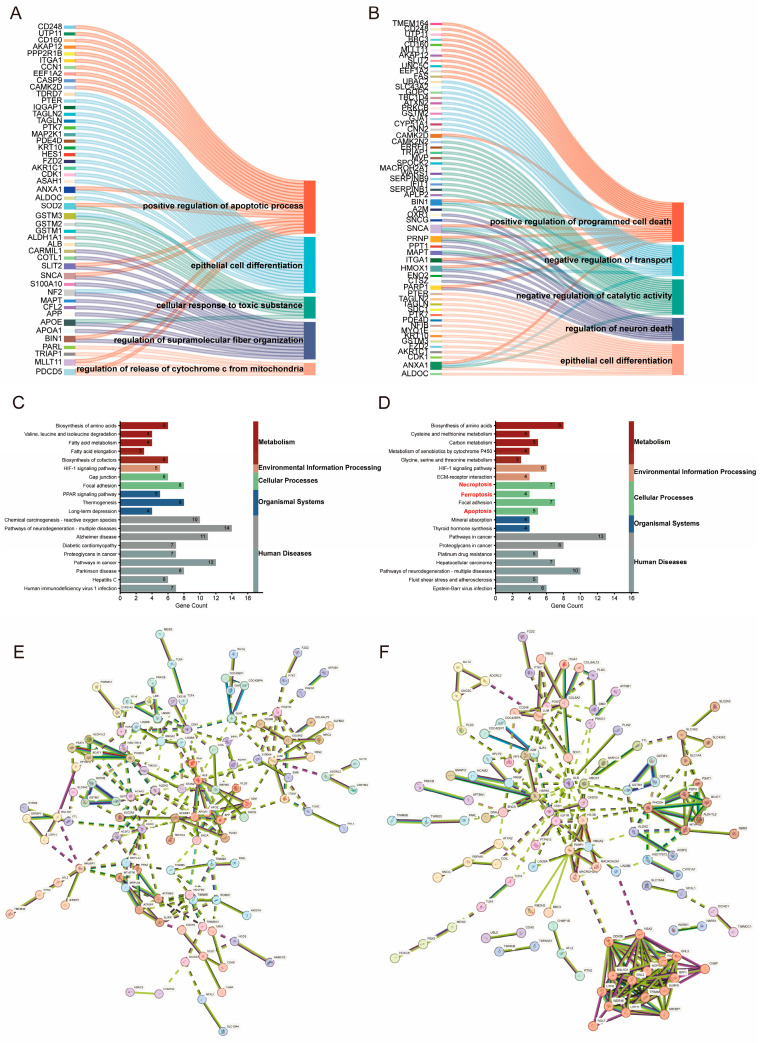
Determination of the modes of FX-induced ovarian cancer cell death by proteomics. (**A**,**B**) GO analysis of changed proteins after FX treatment for 48 h. (**C**,**D**) KEGG analysis of changed proteins after FX treatment for 48 h. (**E**,**F**) The most significant cluster network identified using the MCODE plugin.

To clarify the functional roles and molecular mechanisms of FX treatment in OC, we conducted TMT-based LC-MS/MS analysis on precipitates isolated from A2780 cells. These cells were treated with either a control medium or media supplemented with different concentrations of FX (10 μM or 20 μM). We performed the Gene Ontology (GO) enrichment analysis to understand protein expression changes. As expected, the GO analysis indicated that treatment with 10 μM FX impacted Mitochondrial membrane potential (MMP) and the regulation of reactive oxygen species (ROS), suggesting FX plays a role in modulating mitochondrial homeostasis in ovarian cancer cells (Figure 2A). Treatment with 20 μM FX further influenced programmed cell death pathways, suggesting that FX may induce cell death in OC cells through the regulation of programmed cell death (Figure 2B). Moreover, the Kyoto Encyclopedia of Genes and Genomes (KEGG) analysis showed that different concentrations of FX therapy were connected with specific biological functions. Specifically, treatment with 10 μM FX primarily influenced pathways related to reactive oxygen species, while treatment with 20 μM FX predominantly affected pathways associated with ferroptosis, apoptosis, and necroptosis (Figure 2C,D). A protein–protein interaction (PPI) network was generated by submitting the overlapping core target genes to the STRING database. The resulting network was imported into Cytoscape 3.7.2 for construction and visualization (Figure S2A,B). The MCODE plugin distinguished the PPI network by cluster (Figure 2E,F). These findings suggested that FX, particularly at higher concentrations, has the potential to induce diverse forms of OC cell death, offering insight into its potential application in functional foods for cancer prevention or therapy.

### 2.3. FX-Induced PANoptosis in Ovarian Cancer Cells

**Figure 3:**
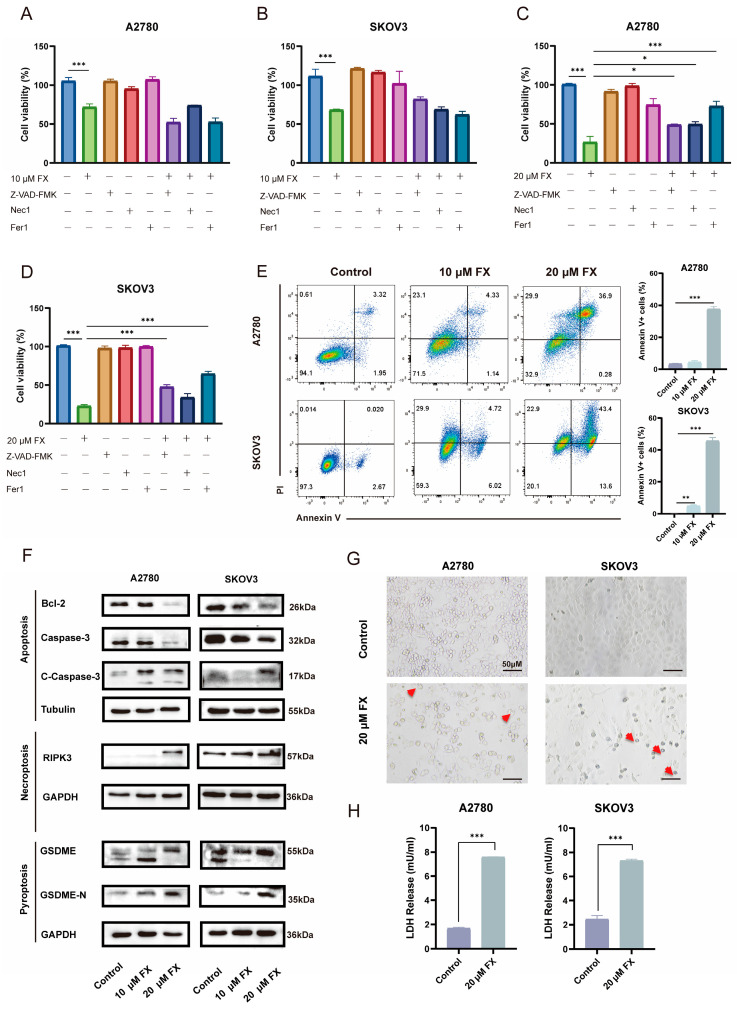
FX-induced programmed cell death in ovarian cancer cells. Inhibitors of apoptosis, ferroptosis, and necrosis co-cultured with 10 μM (**A**,**B**) FX and 20 μM (**C**,**D**) FX to detect ovarian cancer cell viability. (**E**) The apoptosis rate was measured by flow cytometry in A2780 and SKOV3 cells. (**F**) Western blotting analyses were performed in A2780 and SKOV3 cells to measure protein expression. (**G**) Representative photographs of pyroptotic cells are shown. Red arrows represent pyroptotic cells. (**H**) The release of LDH was measured using LDH detection kits. * *p* < 0.05, ** *p* < 0.01, *** *p* < 0.001.

To examine the form of death via which FX leads to OC cell death, A2780 and SKOV3 cells were co-treated with specific inhibitors targeting apoptosis (Z-VAD-FMK), necrosis (Necrostatin-1, Nec-1), and ferroptosis (Ferrostatin-1, Fer-1) for 48 h. FX was administered at concentrations of 10 μM (Figure 3A,B) and 20 μM (Figure 3C,D). The 10 μM FX treatment resulted in the partial induction of cell death in OC cells; however, none of the inhibitors exhibited significant restorative effects. Subsequent cell viability assays revealed that 20 μM FX exerts anti-proliferative effects on A2780 and SKOV3 cells through multiple cell death pathways, including apoptosis, ferroptosis, and necroptosis. To assess apoptosis induction by FX, annexin V and PI staining were employed to quantify the apoptotic rates in treated cells. Marked increases in apoptotic rates were observed following 20 μM FX treatment (Figure 3E). Furthermore, the levels of apoptosis-related proteins, including Bcl2 and cleaved caspase-3, were examined in A2780 and SKOV3 cells following FX treatment. Our findings demonstrated the upregulation of cleaved caspase-3 and the downregulation of the anti-apoptotic protein Bcl2, supporting the apoptotic effects of 20 μM FX treatment. Moreover, WB analysis revealed an increase in the conversion of GSDME and RIPK3 following 20 μM FX treatment in A2780 and SKOV3 cells, indicating enhanced pyroptosis and necroptosis activity (Figure 3F). Significant morphological changes, including cellular swelling and the formation of large bubbles, were observed in A2780 and SKOV3 cells following treatment with 20 μM FX (Figure 3G). Moreover, FX increased the release of lactate dehydrogenase (LDH) (Figure 3H). The results indicate that 10 μM FX treatment induces limited levels of apoptosis. In contrast, 20 μM FX significantly promotes apoptosis, pyroptosis, necroptosis, and ferroptosis. This is also consistent with the results of proteomics. PANoptosis is a form of PCD that integrates pyroptosis, apoptosis, and necroptosis. Our research has demonstrated, for the first time, that FX can induce PANoptosis in tumor cells, offering a unified framework for understanding the interconnected molecular mechanisms behind FX’s anti-tumor effect.

### 2.4. FX-Induced Ferroptosis in OC Cells

**Figure 4:**
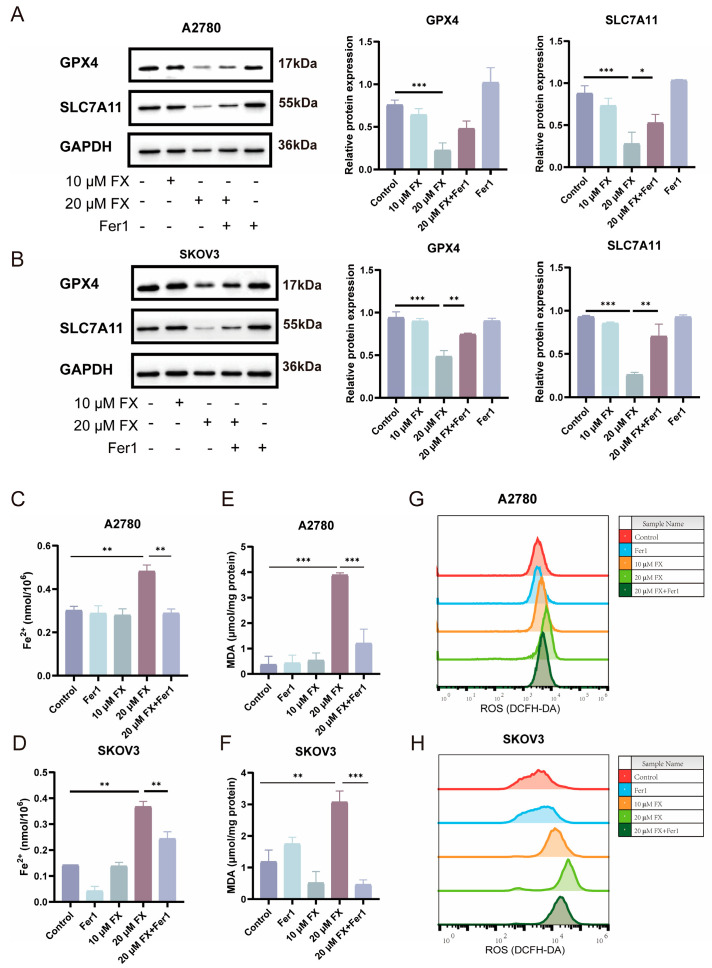
FX-induced ferroptosis in ovarian cancer cells. (**A**,**B**) Western blotting analyses were conducted on A2780 and SKOV3 cells to quantify the protein expression levels of GPX4 and SLC7A11. (**C**,**D**) Fe^2+^ in A2780 and SKOV3 cells was analyzed using commercial detection kits. (**E**,**F**) MDA levels were detected in A2780 and SKOV3 cells, respectively. (**G**,**H**) Intracellular ROS levels of A2780 and SKOV3 cells after the 10 μM and 20 μM FX treatment. * *p* < 0.05, ** *p* < 0.01, *** *p* < 0.001.

Moreover, the apoptosis inhibitor (Z-VAD-FMK) and necroptosis inhibitor specifically restored FX function only in SKOV3 cells, but the ferroptosis inhibitor (Fer1) effectively reversed FX-induced cell death both in A2780 and SKOV3 cells. We examined various forms of FX-induced programmed cell death in OC cells and identified ferroptosis as the predominant mechanism, prompting us to focus our investigation on this pathway. We comprehensively investigated the effects of FX on ferroptosis in OC cells. A2780 and SKOV3 cells were divided into several treatment groups as follows: FX (10 and 20 μM), Fer-1 (1 μM), and FX combined with Fer-1 (20 μM FX + 1 μM Fer-1), aiming to assess the induction of ferroptosis by FX. Following FX treatment, the ferroptosis-related protein GPX4 and SLC7A11 levels in both A2780 and SKOV3 cells were significantly reduced (Figure 4A,B). Notably, Fer1 co-treatment partially restored GPX4 expression in both cell lines, with statistical significance achieved in SKOV3 (*p* < 0.05) and a consistent trend observed in A2780. Additionally, we assessed the intracellular Fe^2+^ levels and observed an increase following FX treatment, which was significantly attenuated by Fer-1 (Figure 4C,D). To investigate the influence of lipid peroxidation on ferroptosis, we measured the concentrations of malondialdehyde (MDA), the end result of lipid peroxidation, in A2780 and SKOV3 cells after they were treated with FX. Consistently, we noticed a significant rise in MDA levels when exposed to FX, although Fer-1 significantly reduced this increase (Figure 4E,F). Moreover, we quantified intracellular ROS accumulation using flow cytometry. The results demonstrated a significant increase in ROS levels in A2780 and SKOV3 cells after FX treatment (Figure 4G,H). Taken together, FX can induce ovarian cancer cells ferroptosis. Since 20 µM FX significantly induced ferroptosis in A2780 and SKOV3 cells, all subsequent experiments were conducted and studied using 20 µM FX.

### 2.5. FX-Induced Ferroptosis in Ovarian Cancer Cells Is Associated with Mitochondrial Dysfunction

**Figure 5:**
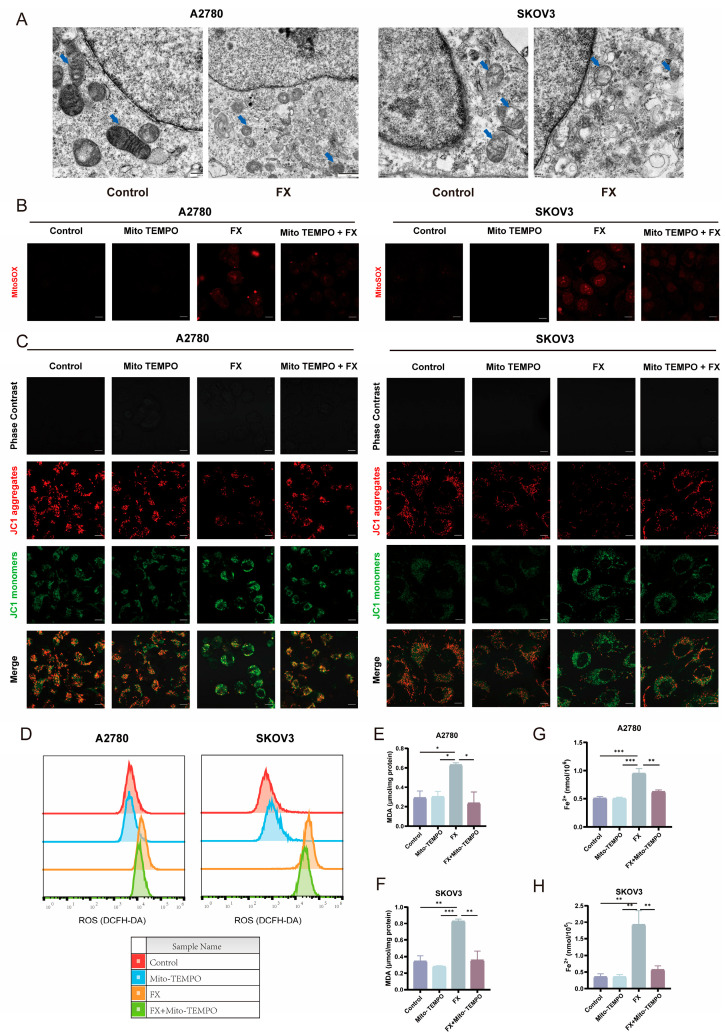
FX-induced ferroptosis in OC cells is associated with mitochondrial dysfunction. (**A**) TEM was used to observe the ultrastructure of the mitochondria in OC cells. The blue arrows point to the mitochondria in OC cells. (**B**) Evaluation of mitochondrial ROS levels in OC cells using MitoSOX Red. (**C**) Intracellular fluorescence of JC-1 in OC cells for the detection of MMP. (**D**) Intracellular ROS levels of OC cells. (**E**,**F**): MDA levels were detected in OC cells, respectively. (**G**,**H**): Fe^2+^ in OC cells was analyzed using commercial detection kits. Scale bar = 20 μm. * *p* < 0.05, ** *p* < 0.01, *** *p* < 0.001 vs. control group.

Mitochondria have a crucial function in several types of cell death, such as apoptosis [35], ferroptosis [36], pyroptosis [37], and necroptosis [38]. Mitochondria serve as crucial cellular energy sources and are central ferroptosis regulators. We assessed mitochondrial ultrastructure, mitochondrial morphology, and MMP. We utilized transmission electron microscopy (TEM) to examine mitochondrial ultrastructure (Figure 5A). FX consistently led to reduced mitochondrial size, double membrane thickness, decreased mitochondrial cristae, and outer membrane rupture in both A2780 and SKOV3 cells. Recent studies underscore the essential role of mitochondria-mediated ROS production in initiating lipid peroxidation and the subsequent ferroptosis [36,39]. Furthermore, impaired mitochondria have been identified as the primary source of ROS, a critical indicator of ferroptosis [40]. We utilized the mitochondrial ROS inhibitor Mito TEMPO to investigate the mechanism of ferroptosis induced by FX. A2780 and SKOV3 cells were treated under various experimental conditions as follows: FX group (20 μM FX), Mito TEMPO group (10 μM Mito TEMPO), and FX and Mito TEMPO group. Mitochondrial ROS was evaluated by the MitoSOX Red fluorescent probe (Figure 5B). MMP was quantified utilizing the JC-1 fluorescent probe, with the red-to-green fluorescence intensity ratio indicative of mitochondrial depolarization. Elevated ROS levels can induce the mitochondrial membrane permeability transition pore (MPT) to open, which in turn leads to a reduction in mitochondrial transmembrane potential (Δψm) [41]. Notably, both A2780 and SKOV3 cells exhibited a significant decrease in MMP following FX treatment, which was rescued by Mito TEMPO (Figure 5C). Furthermore, compared with FX treatment, treatment with Mito TEMPO; inhibited the increase in ROS levels (Figure 5D), MDA content (Figure 5E,F), and iron ion accumulation (Figure 5G,H), underscoring the role of FX in mediating the massive release of mitochondrial ROS via mitochondrial damage in ferroptosis. These findings collectively indicate that FX-induced ferroptosis is linked to ROS-mediated mitochondrial dysfunction in OC.

### 2.6. Fucoxanthin-Induced Ferroptosis via the AMPK/Nrf2/HMOX-1 Pathway in OC Cells

To elucidate the mechanism of FX-induced ferroptosis, we further identified differently expressed proteins (DEPs) in FX-treated cells vs. normal cells by proteomics. Compared to the control group, treatment with 10 μM FX led to the upregulation of 155 proteins and the downregulation of 97 proteins in A2780 cells (Figure S3A). Similarly, exposure to 20 μM FX led to the upregulation of 145 proteins and the downregulation of 63 proteins in A2780 cells relative to the control group (Figure S3B). Additionally, proteomic heatmaps revealed the significant upregulation of HMOX1 and SLC3A2 following FX treatment in A2780 cells (Figure S3C). Combined with the KEGG results, we found essential proteins related to ferroptosis in the PPI network. Notably, HMOX1, SLC3A2, AKR1C1, and FTL were among the interacting proteins associated with the ferroptosis pathway (Figure S3D). We employed qRT-PCR to evaluate the expression levels of HMOX1, SLC3A2, AKR1C1, and FTL. Our investigation showed a notable increase in the mRNA levels of HMOX1, SLC3A2, AKR1C1, and FTL in both A2780 and SKOV3 cells following FX treatment (Figure S3E,F). Moreover, an analysis of the GEPIA database indicated the significantly lower expression of HMOX1 in OC tissues compared with normal tissues (Figure S3G). Studies have demonstrated that elevated HMOX1 levels are associated with ferroptosis induction, as observed with erastin [42,43]. Additionally, there is a possibility that there is an increased expression of the HMOX1 gene in OC cells treated with olaparib-Ga, which may be attributed to the heightened sensitivity to Fe^2+^-induced ROS [44].

**Figure 6:**
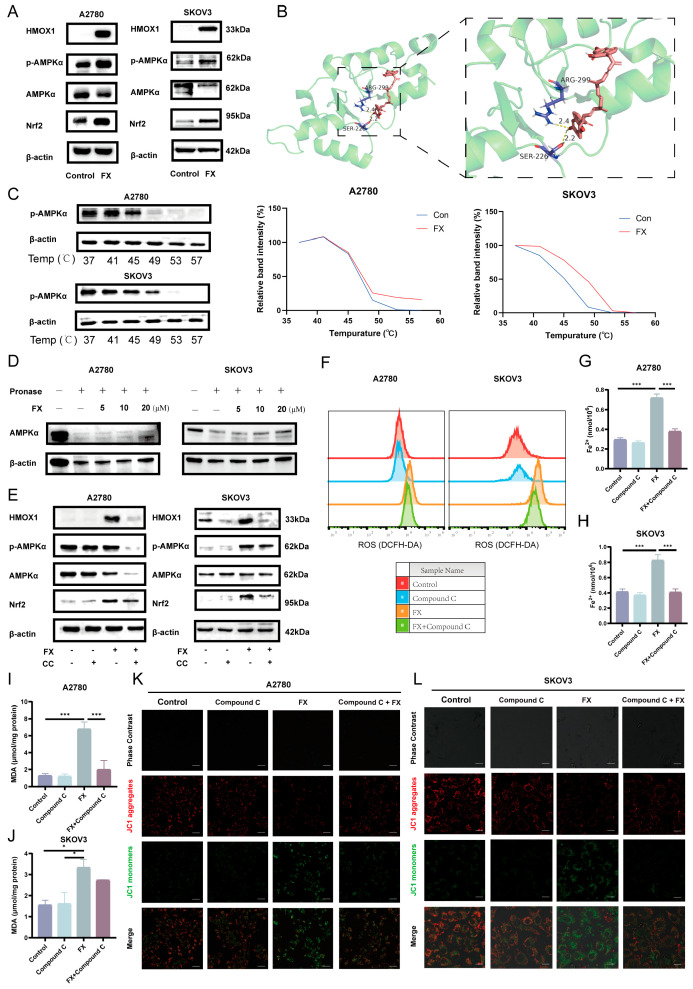
AMPK inhibition can reverse the FX-induced ferroptosis of OC cells. (**A**) The expression of p-AMPK, AMPK, Nrf2, and HMOX1 in A2780 and SKOV3 cells. (**B**) The interaction between FX and AMPK was predicted by molecular docking. (**C**) CETSA showed AMPK target engagement by FX in ovarian cancer cells. (**D**) The protein expression of AMPK of DARTS. Treatment with pronase was conducted for 30 min. Western blot results using anti-AMPK and anti-actin antibodies. The DARTS assay shows that FX protects AMPK, but not actin, from degradation. (**E**) The expression of p-AMPK, AMPK, Nrf2, and HMOX1 in OC cells, subjected to FX and Compound C (CC). (**F**) Intracellular ROS levels of OC cells after FX and CC treatment. (**G**,**H**) Fe^2+^ levels were detected in OC cells, respectively. (**I**,**J**) MDA was analyzed using commercial detection kits. (**K**,**L**) Intracellular fluorescence of JC-1 in OC cells for the detection of MMP. Scale bar = 20 μm. * *p* < 0.05, *** *p* < 0.001 vs. control group.

We propose that HMOX1 plays a pivotal role in FX-regulated ferroptosis in ovarian cancer. Recent studies have shown that vitamin C enhances the susceptibility of pancreatic cancer cells to erastin-induced ferroptosis through the activation of the AMPK/Nrf2/HMOX1 pathway [45]. The AMP-activated protein kinase (AMPK) is a versatile molecule responsive to stress and is pivotal in cell metabolism, survival, and antioxidant responses [46]. Significantly, Nrf2 is directly modulated by AMPK; upon phosphorylation, Nrf2 promotes the synthesis of various antioxidative compounds [47]. Nrf2 functions as a transcription factor that translocates to the nucleus, where it activates the expression of target genes, including HMOX1. Based on these findings, we hypothesize that FX may induce ferroptosis in OC through the AMPK/Nrf2/HMOX1 axis. Western blotting experiments revealed the significant upregulation of p-AMPK, Nrf2, and HMOX1 expression upon treatment with FX (Figure 6A and Figure S4).

FX and AMPK exhibited a robust binding affinity, as shown by the molecular docking experiments (Figure 6B). In order to verify the accuracy of the docking data, we used a cellular thermal shift assay (CETSA) to evaluate the stability of the AMPK inside cells when it interacts with FX. The CETSA findings revealed a negative correlation between temperature and AMPK protein levels, with a notable reduction. However, this fall was notably mitigated in cells treated with FX, as seen in Figure 6C and Figure S5. This suggests that FX therapy successfully shields AMPK from degradation caused by temperature fluctuations. Similarly, the results from DARTS indicated the obvious inhibition of AMPK degradation caused by proteases upon treatment with FX (Figure 6D). In order to explore the impact of FX-induced ferroptosis on OC cells through the AMPK/Nrf2/HMOX1 axis, we used the AMPK inhibitor Compound C (CC). To assess whether FX-induced cytotoxicity depends on AMPK activation, we treated OC cells with FX (20 μM), CC (1 μM for A2780; 2 μM for SKOV3), or their combination for 48 h. MTT assays revealed that FX alone significantly reduced cell viability, whereas CC alone exhibited no intrinsic cytotoxicity. Notably, co-treatment with FX and CC partially restored FX induced cell death (Figure S6), suggesting that AMPK inhibition attenuates the cytotoxic effects of FX alone. Western blotting experiments revealed the significant downregulation of Nrf2 and HMOX1 expression upon treatment with both FX and CC (Figure 6E and Figure S7). Considering that the buildup of ROS and lipid peroxidation are distinctive characteristics of ferroptosis, we next examined the impact of AMPK inhibition on these parameters. The inhibition of AMPK resulted in a reduction in ROS content and lipid peroxidation levels (Figure 6F). Furthermore, Fe^2+^ levels were measured using a colorimetric assay kit, showing an increase following FX treatment, which was mitigated by CC (Figure 6G,H). Consistently, a decrease in malondialdehyde (MDA) levels, indicative of reduced lipid peroxidation, was observed with CC treatment, contrasting with the elevated MDA levels upon FX treatment (Figure 6I,J). Notably, FX treatment led to a marked decrease in MMP in both A2780 and SKOV3 cells, an effect that was partially restored by CC (Figure 6K,L). This suggested that AMPK is a direct target of FX-induced ferroptosis in OC cells.

**Figure 7:**
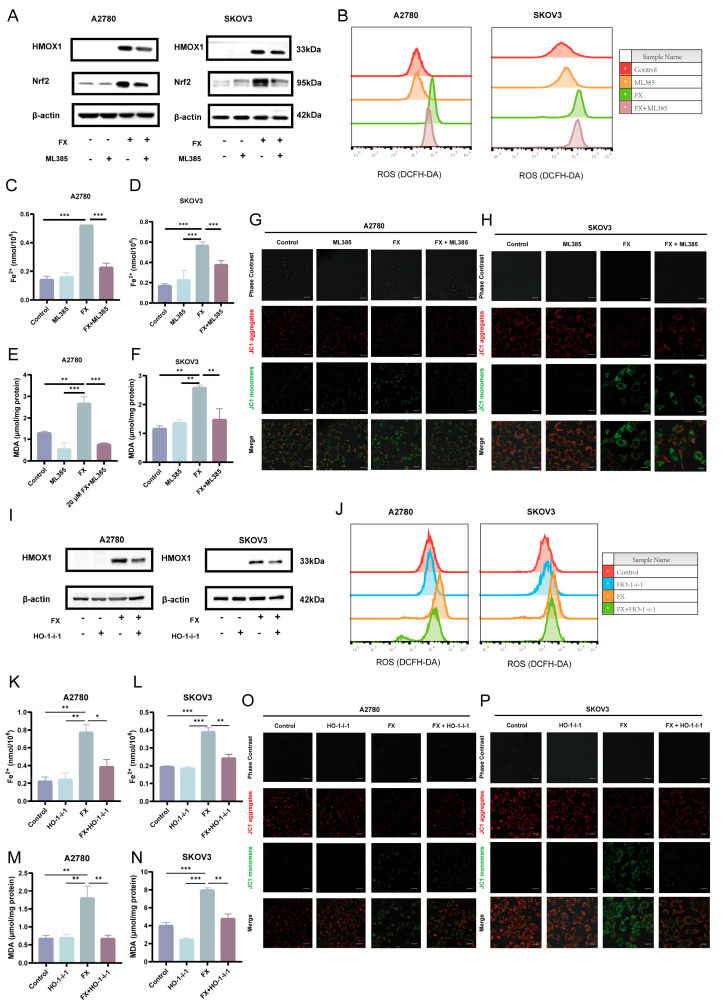
Nrf2 inhibition and HMOX1 inhibition can reverse the FX-induced ferroptosis of OC cells. (**A**) The levels of Nrf2 and HMOX1 in A2780 and SKOV3 cells, subjected to FX and ML385. (**B**) Intracellular ROS levels of OC cells after FX and ML385 treatment. (**C**,**D**) Fe^2+^ expression was detected in A2780 and SKOV3 cells, respectively. (**E**,**F**) MDA was analyzed using commercial detection kits. (**G**,**H**) Intracellular fluorescence of JC-1 in OC cells for the detection of MMP. (**I**) The levels of HMOX1 in A2780 and SKOV3 cells, subjected to FX and HO-1-i-1. (**J**) Intracellular ROS levels of OC cells after FX and HO-1-i-1 treatment. (**K**,**L**) Fe^2+^ expression was detected in A2780 and SKOV3 cells, respectively. (**M**,**N**) MDA was analyzed using commercial detection kits. (**O**,**P**) Intracellular fluorescence of JC-1 in OC cells for the detection of MMP. Scale bar = 20 μm. * *p* < 0.05, ** *p* < 0.01, *** *p* < 0.001 vs. control group.

It has been reported that AMPK activation facilitates the nuclear accumulation of Nrf2 [48]. Nrf2 is pivotal in regulating the antioxidant system, modulating oxidative stress by controlling HMOX1 expression, and influencing intracellular heme production and degradation [49,50]. And Nrf2 may exert opposing pro- or anti-ferroptosis effects depending on the cellular context [51]. To examine the involvement of Nrf2 in ferroptosis triggered by FX in OC cells, we utilized the Nrf2 inhibitor ML-385. OC cells were treated with FX (20 μM) and ML-385 (1 μM) to assess their effects on Nrf2 and HMOX1 expression. Western blot analysis demonstrated that both FX and ML-385 effectively reduced the expression levels of Nrf2 and HMOX1, as depicted in Figure 7A and Figure S8. We quantified the accumulation of ROS and lipid peroxidation, which are established indicators of ferroptosis. Our findings demonstrated that the inhibition of Nrf2 resulted in decreased levels of ROS and lipid peroxidation, as illustrated in Figure 7B. Additionally, we measured Fe^2+^ levels using a specific colorimetric assay kit, revealing an increase in Fe^2+^ levels following FX treatment. While ML-385 could call back the levels of Fe^2+^ (Figure 7C,D). Consistently, treatment with FX increased malondialdehyde (MDA) levels, indicating raised lipid peroxidation. In contrast, ML385 treatment led to reduced MDA levels (Figure 7E,F). Additionally, FX treatment led to a significant reduction in MMP in both A2780 and SKOV3 cells, an effect that was reversed by ML-385, highlighting the role of FX-induced mitochondrial damage in ferroptosis (Figure 7G,H).

We used the selective HMOX1 inhibitor HO-1-i-1 to examine the function of HMOX1 in FX-induced ferroptosis in OC cells. OC cells were treated with FX and HO-1-i-1 to assess their impact on HMOX1 expression. Western blot examination revealed that HO-1-i-1 markedly inhibited FX-increased HMOX1 expression levels (Figure 7I). Additionally, we evaluated the increase in ROS and lipid peroxidation, which are essential indicators of ferroptosis. Our results demonstrated that the suppression of HMOX1 correlated with decreased levels of ROS and lipid peroxidation (Figure 7J). To clarify FX-induced ferroptosis, we assessed Fe^2+^ concentrations, which indicated an elevation in Fe^2+^ after FX treatment, while HO-1-i-1 mitigated this increase (Figure 7K,L). FX treatment consistently increased MDA levels, whereas HO-1-i-1 administration was associated with decreased MDA levels (Figure 7M,N). Furthermore, both A2780 and SKOV3 cells demonstrated a significant reduction in MMP after FX treatment. This effect was mitigated by HO-1-i-1, underscoring the role of FX-induced mitochondrial dysfunction in ferroptosis (Figure 7O,P).

### 2.7. FX-Induced Ferroptosis in Ovarian Cancer and Suppressed Tumor Growth in a Mouse Model

**Figure 8:**
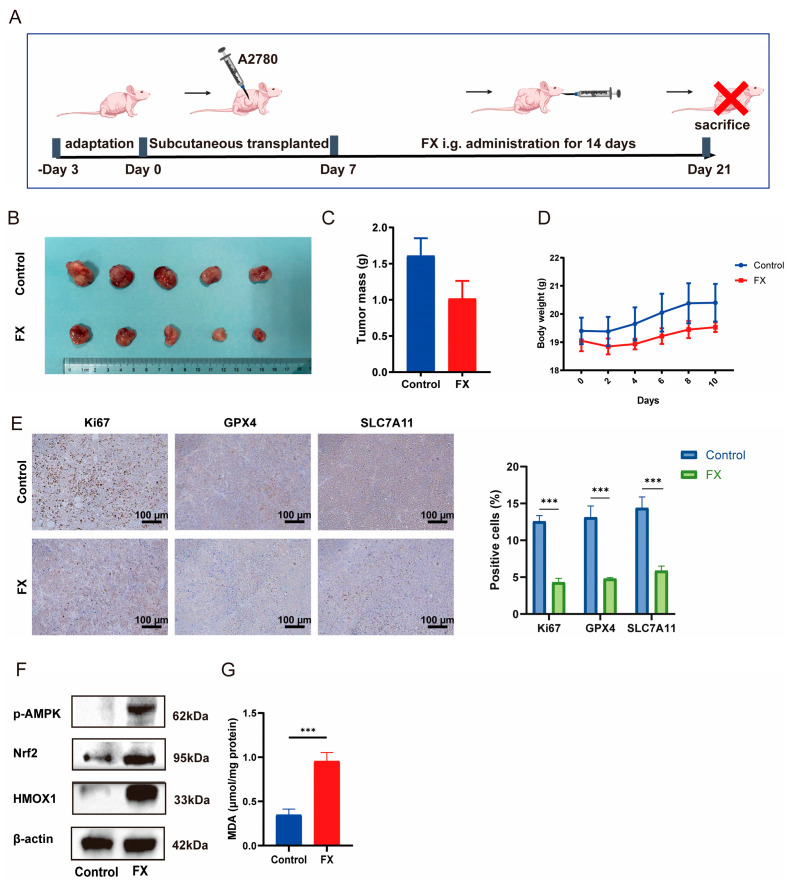
FX suppresses tumor growth in a mouse model. (**A**) Timeline depicting the experimental timetable for A2780-xenografted nude mice. (**B**) Mice tumors. (**C**) Volumes of tumors in A2780-xenografted nude mice after FX (100 mg/kg) treatment. (**D**) Body weights of the mice. (**E**) Immunohistochemical techniques were examined to assess the levels of the proliferation marker Ki67 and the levels of SLC7A11, and GPX4. (**F**) Western blot of critical target expressions of HMOX1, p-AMPK, and Nrf2. (**G**) The expression levels of MDA in malignancies were analyzed. *** *p* < 0.001 vs. control group.

To investigate the relationship between FX-mediated suppression of ovarian cancer (OC) tumor growth and ferroptosis induction, we subcutaneously implanted A2780 cells into the right flank of nude mice. After a 3-day incubation period, mice were given repeated doses of 100 mg/kg FX or an equal amount of saline orally for 14 days (Figure 8A). Remarkably, FX-treated groups exhibited significant reductions in tumor volume and weight compared to the vehicle-treated group, as illustrated in Figure 8B,C. There was no significant difference in body weight between the FX-treated and control groups of mice (Figure 8D). Notably, the percentage of Ki-67-positive cells decreased notably after FX treatment, which indicates that FX effectively inhibits tumor development in vivo (Figure 8E). To investigate the relationship between the FX-mediated suppression of tumor development and ferroptosis, we conducted an immunohistochemistry (IHC) analysis to assess the expression of vital ferroptosis-associated targets, including SLC7A11 and GPX4 in tumor tissues (Figure 8E). Western blot analysis revealed that FX markedly upregulated *p*-AMPK, Nrf2, and HMOX1 expression (Figure 8F and Figure S9). Additionally, there was a notable increase in MDA levels in FX-treated tumors, as depicted in Figure 8G. These findings suggest that FX-induced ferroptosis contributes to the reduction in tumor development in vivo.

## 3. Discussion

While platinum-based regimens and molecular-targeted agents have reshaped the clinical management of ovarian cancer, the therapeutic landscape remains constrained by the following two persistent challenges: acquired chemoresistance in >70% of patients within 2 years [3] and the dose-limiting toxicities of existing therapies [2]. This therapeutic impasse has stimulated the growing exploration of marine-derived bioactive compounds, which exhibit unique structural diversity and mechanism profiles compared to terrestrial sources. For instance, trabectedin, isolated from the tunicate *Ecteinascidia turbinata*, has demonstrated clinical efficacy in platinum-sensitive recurrent ovarian cancer by selectively targeting DNA repair pathways and tumor-associated macrophages [52]. Similarly, bryostatin-1 from the bryozoan *Bugula neritina* inhibits metastasis through the PKC-mediated downregulation of MMP-9 and VEGF [53]. Additionally, plitidepsin, a cyclic depsipeptide from the Mediterranean ascidian *Aplidium albicans*, synergizes with platinum agents by inducing oxidative stress and apoptosis in resistant cells [54]. These marine-derived agents collectively highlight the untapped potential of oceanic resources in overcoming conventional therapeutic limitations in OC.

Marine-derived compounds have emerged as a valuable source of bioactive molecules with significant anticancer potential. Marine-derived compounds have significantly contributed to the development of anticancer agents. In recent years, six marine-derived compounds have received clinical approval as anticancer drugs. Among them, four—Lurbinectedin, *Polatuzumab vedotin*, *Enfortumab vedotin*, and *Belantamab mafodotin*—have been authorized by the FDA and/or EMA for the treatment of ovarian cancer, breast cancer, urothelial cancer, and multiple myeloma, respectively [55]. These examples underscore the potential of marine organisms in providing novel compounds for cancer therapy. Fucoxanthin is present in *Laminaria japonica*, *Undaria pinnatifida*, and some diatoms and has significant biological characteristics. Studies have shown that FX exhibits potent anticancer properties by apoptosis induction, cell cycle arrest, and EMT inhibition [56,57]. While recent investigations have found that FX can inhibit OC cells growth through deactivating the STAT3/c-Myc pathway [34]. It has been shown that FX, in combination with DOX, can induce apoptosis and overcome multidrug resistance in ovarian cancer cells [33], but its specific action mechanisms remain ambiguous and need further exploration.

We analyzed the multiple ways FX causes OC cell death by methods such as proteomics and molecular biology. The results revealed that FX induces programmed cell death. PCD encompasses various forms, primarily including apoptosis, autophagy, and necroptosis. FX has been shown to activate the intrinsic apoptotic pathway in various cancer types, including breast, colon, and prostate cancer, by modulating the Bcl-2 family of proteins and caspase activation [58,59]. Additionally, FX has been reported to induce autophagy, a process that promotes cell survival under stress but can also lead to cell death under certain conditions [60]. However, the role of FX in other forms of PCD, such as necroptosis, pyroptosis, and ferroptosis, has remained largely unexplored until now. PANoptosis, a newly discovered form of regulated cell death, integrates key molecular components of pyroptosis, apoptosis, and necroptosis, activating these three death pathways simultaneously to achieve synergistic multi-mechanistic killing [61]. Within the tumor microenvironment, PANoptosis plays a crucial role in regulating tumor cell survival and death by modulating inflammatory responses and antitumor immunity [62]. Our study shows for the first time that FX can induce PANoptosis in cancer cells, offering a unified framework to understand the interconnected molecular mechanisms behind FX’s anti-tumor effect. FX-treated cells showed the downregulation of Bcl-2 and an upregulation of cleaved caspase-3, suggesting that FX promotes apoptosis by tipping the balance towards pro-apoptotic signaling. This is consistent with previous studies that have implicated apoptosis as a key mechanism in tumor suppression [63]. Necroptosis is regulated necrosis mediated by the RIPK3 and its downstream effector, mixed lineage kinase domain-like pseudokinase (MLKL). RIPK3 activation leads to the phosphorylation of MLKL, which then translocates to the plasma membrane, causing membrane rupture and cell death [64]. Our data showing the upregulation of RIPK3 in FX-treated cells suggest that necroptosis is also a key component of the PANoptotic response. Gasdermin E (GSDME), a member of the gasdermin family, is a critical executor of pyroptosis. Upon cleavage by caspase-3, GSDME forms pores in the plasma membrane, leading to cell swelling and eventual lysis [65]. Our observation of GSDME activation in FX-treated cells indicates that pyroptosis is a significant component of the PANoptotic response induced by FX. Our study provides novel insights into the mechanism of action of FX, demonstrating its ability to induce PANoptosis in tumor cells. This is particularly significant given the growing interest in targeting multiple cell death pathways to overcome therapy resistance in cancer. The simultaneous modulation of apoptosis, pyroptosis, and necroptosis by FX suggests that it may be a promising candidate for further development as an anticancer agent. Moreover, our findings contribute to the broader understanding of PANoptosis as a convergent mechanism for tumor cell elimination, which may have implications for the design of future cancer therapies.

Furthermore, we first discovered that ferroptosis plays a pivotal role in FX-induced OC cell death. Our data demonstrated that FX markedly downregulated GPX4 and SLC7A11 protein expression, upregulated Fe^2+^ levels, MDA content, and ROS levels in OC cells. We examined various forms of FX-induced PCD in OC cells and identified ferroptosis as the predominant mechanism, prompting us to further investigate its mechanism. Typical ferroptosis features, such as excessive iron accumulation and lipid oxidation, were observed in OC, accompanied by mitochondrial dysfunction. This included alterations in MMP and increased production of mt-ROS. Mitochondria serve as crucial cellular energy sources and participate in signaling pathways that influence both physiological and pathological processes [66,67,68]. Mitochondria have a crucial function in ferroptosis [36]. Our electron microscopy results substantiate that mitochondria undergo distinct morphological alterations during ferroptosis. Recent studies underscore the essential role of mitochondria-mediated ROS production in initiating lipid peroxidation and the subsequent ferroptosis [36]. Our findings further suggest that FX promotes mitochondrial ROS accumulation, implicating mitochondrial ROS generation as a mechanism of FX-induced ferroptosis.

Based on proteomic evidence, we are the first to suggest that FX activates the AMPK/Nrf2/HMOX1 pathway, as this axis has previously been implicated in ferroptosis induction by erastin and vitamin C agents in pancreatic cancer cells [45]. Our study is the first to establish a direct link between FX and this pathway and underscore the functional relevance of this axis in FX-mediated ferroptosis. We confirmed that FX directly activates AMPK. AMPK, a master regulator of cellular energy homeostasis, plays a pivotal role in ferroptosis. The AMPK-mediated phosphorylation of Beclin1 has been shown to potentiate ferroptosis by inhibiting system Xc− activity and cysteine uptake [69]. Then, we found that AMPK activation by FX facilitates the nuclear accumulation of Nrf2. The genetic depletion of AMPK in mouse embryonic fibroblasts significantly reduces Nrf2-dependent HMOX1 expression, highlighting the regulatory significance of the AMPK/Nrf2/HMOX1 axis in ferroptosis [70]. Nrf2 and its downstream target HMOX1 are critical mediators of FX-induced ferroptosis. HMOX1, an enzyme that degrades heme to produce free iron, has emerged as a key player in ferroptosis induction across various cancer types. For example, withaferin A induces ferroptosis in neuroblastoma cells via enhanced iron accumulation and ROS production mediated by HMOX1 [71]. Polyphyllin I suppressed hepatocellular carcinoma progression by triggering ferroptosis through mitochondrial dysfunction through the Nrf2/HMOX1/GPX4 axis [72]. Our data corroborate these findings, showing that FX significantly upregulates HMOX1 expression in a manner that is dependent on AMPK and Nrf2. The ability of FX to simultaneously activate AMPK, Nrf2, and HMOX1 distinguishes it from other ferroptosis inducers. This multifaceted mechanism of action may enhance the therapeutic efficacy of FX, particularly in cancers resistant to conventional ferroptosis inducers.

Our in vivo study confirmed that the oral administration of FX effectively suppressed OC tumor growth without significant weight loss, consistent with previous reports supporting its safety profile. IHC analysis revealed the altered expression of key ferroptosis markers, including SLC7A11 and GPX4, while Western blot results demonstrated that FX upregulated p-AMPK, Nrf2, and HMOX1, indicating its role in ferroptosis regulation. Collectively, our findings highlight FX as a promising marine-derived therapeutic agent for OC, with ferroptosis induction serving as a pivotal mechanism underlying its antitumor activity. This study is the first to reveal that the mechanism underlying the antitumor effects of FX in vivo is associated with ferroptosis.

## 4. Materials and Methods

### 4.1. Cell Lines and Cell Culture

A2780 and SKOV3 cells were obtained from the Cell Bank of Type Culture Collection of the Chinese Academy of Sciences. A2780 and SKOV3 cells were cultured in DMEM (Gibco, Grand Island, NY, USA), supplemented with the addition of 10% fetal bovine serum (FBS). Both cell lines were cultured in a 5% CO_2_ incubator at 37 °C.

### 4.2. Reagents

Z-VAD-FMK (HY-16658B), Necrostatin-1 (HY-15760), Ferrostatin-1 (HY-100579), Mito-TEMPO (HY-112879), ML385 (HY-100523), Dorsomorphin (Compound C, HY-13418A), Heme Oxygenase-1-IN-1 (HY-111798), and MitoSOX Red (HY-D1055) were purchased from Medchem Express (Monmouth Junction, NJ, USA). We obtained FX according to a previously described method [28]. The stock solution was diluted with a cell culture medium to obtain the required concentration before application.

### 4.3. Cell Viability Assay

The cytotoxicity of FX on OC cells was assessed via the MTT assay. A configuration of 8 × 10^3^ cells per well was placed on a 96-well culture plate (Corning, NY, USA) and left to attach for 24 h. Following adhesion, the cells were incubated for 24, 48, and 72 h with varying final concentrations of FX (0, 2.5, 5, 10, 15, 20, 40, 60, 80, and 100 μM).

### 4.4. Colony Formation Assay

A2780 and SKOV3 cells were seeded in 6-well plates from a cell culture flask at densities of 600 and 800 cells per well, respectively. After allowing 24 h for adherence, the cells were cultured with FX (0, 10, and 20 μM). Each well was filled with 1 mL of 4% paraformaldehyde and fixed for 15 min. Cells were then stained with 1% crystal violet (Beyotime, Haimen, Jiangsu, China) at room temperature for 15 min. Colonies with over 50 cells were photographed, counted, and analyzed.

### 4.5. Migration and Invasion Assays

For the wound healing test, 3 × 10^5^ OC cells (A2780, SKOV3) were injected onto a 6-well plate and subsequently scratched at the center of the wells using a 10 μL pipette tip with or without FX treatment, and they were then cultivated in serum-free media. Images were captured at 0 and 48 h. For the transwell assay, 700 µL of medium supplemented with 20% FBS was added to the lower well of each chamber. The membrane was coated with Matrigel before the addition of the cells to the top inserts. Subsequently, 1 × 10^5^ cells suspended in serum-free media were introduced into the top inserts. Upon the completion of the designated time of incubation, the total quantity of cells adhering to the membrane’s bottom surface was evaluated.

### 4.6. Proteomics

The proteins detected in each independent sample of the FX group and the control group were subjected to bioinformatics analysis. When selecting differentially expressed proteins, the protein samples should exhibit a fold change of 1.2 or greater between the control and FX groups, and statistical significance should be determined using paired two-tailed Student’s *t*-test, with a *p*-value less than 0.05. Gene Ontology (GO) enrichment and Kyoto Encyclopedia of Genes and Genomes (KEGG) pathway analysis were conducted using the STRING database (https://string-db.org/cgi/input (accessed on 28 December 2023)), with all identified proteins serving as background references. The significance level was set at *p* < 0.05.

### 4.7. Apoptosis Analysis

The detection of apoptosis was carried out through Annexin V-FITC/PI double staining. Apoptosis was evaluated by flow cytometry at 488 nm, with each analysis using 2 × 10^4^ cells following the addition of 200 μL binding buffer. Data analysis was performed using FlowJo 10.

### 4.8. Western Blot Analysis

The proteins were obtained from cells or tumor tissues, and a Western blot analysis was performed following the methods described in a recent work [10]. Primary antibodies against GPX4 (13116), SLC7A11 (5738), GAPDH (4249), and actin (2548) were purchased from Cell Signaling Technology (Danvers, MA, USA). HMOX-1 (T55113), AMPK and phospho-AMPK alpha (Thr172) (TA3423), and Nrf2 (T55136) were purchased from Abmart. GAPDH (ab8245), Bcl-2 (ab182858), LC3-I/II (ab48394), caspase-3 (ab32351), and tubulin (ab179513) were purchased from Abcam (Waltham, MA, USA).

### 4.9. Measurement of Fe^2+^

The cellular Fe^2+^ level was determined using the Ferrous Iron Colorimetric Assay Kit (E-BC-K881-M, Elabscience, Wuhan, China). The tissue sample was utilized to quantify Fe^2+^ concentrations using an assay kit (E-BC-K773-M, Elabscience, Wuhan, China) in accordance with the manufacturer’s instructions.

### 4.10. Measurement of Malondialdehyde (MDA)

The cellular content of MDA was measured using the cell MDA assay kit (Beyotime, Haimen, Jiangsu, China), which employs the reactivity of thiobarbituric acid (TBA).

### 4.11. Measurement of ROS

According to the manufacturer’s guidelines, ROS levels were quantified using DCFH-DA (Beyotime, Haimen, Jiangsu, China).

### 4.12. Transmission Electron Microscopy (TEM)

The TEM technique was used to assess the detailed structure of mitochondria. We utilized a Hitachi HT7700 transmission electron microscope to conduct observations and capture very detailed images of the ultrastructure.

### 4.13. Mitochondrial Membrane Potential

The MitoProbe™ JC-1 test kit (Beyotime, Cat. No. C2006) was used to assess mitochondrial membrane potential (MMP), according to the instructions provided by the manufacturer. Fluorescence intensity was quantified with a laser confocal microscope. JC-1 molecules form J-aggregates, emitting red fluorescence at 555 nm in normally functioning mitochondria. In mitochondria with reduced membrane potential, JC-1 exists as individual J-monomers emitting green fluorescence at 488 nm.

### 4.14. MitoSOX Red Staining

MitoSOX Red was prepared in DMSO to a final concentration of 5 mM and stored at −20 °C. Treated cells were labeled with MitoSOX Red and incubated for 15 min. They were then washed 3 times with PBS. The acquisition of images was performed with a laser confocal microscope.

### 4.15. Real-Time Quantitative PCR Analysis

The TRIzol method was employed to isolate the total RNA from cells. The RNA was transcribed reversely using Hifair^®^ III 1st Strand cDNA Synthesis SuperMix for qPCR (11137ES60, Yeasen, Shanghai, China). The real-time polymerase chain reaction (PCR) was conducted using the Hieff UNICON^®^ Universal Blue qPCR SYBR Green Master Mix (11184ES08, Yeasen). The amounts of GAPDH mRNA were utilized as an internal control. The mRNA expression values were determined using the 2^−ΔΔCt^ methods.

The primers are listed as follows:

HMOX1: 5′-AAGACTGCGTTCCTGCTCAAC-3′ (forward);

5′-AAGCCCTACAGCAACTGTCG-3′ (reverse);

AKR1C1: 5′- GCCATATTGATTCTGCTCATTTAT-3′ (forward);

5′-TGGGAATTGCTCCAAAGC-3′ (reverse);

SLC3A2: 5′-CCAGAAGGATGATGTCGCTCAG-3′ (forward);

5′-GAGTAAGGTCCAGAATGACACGG-3′ (reverse);

FTL: 5′-AGCGTCTCCTGAAGATGCAA-3′ (forward);

5′-CAGCTGGCTTCTTGATGTCC-3′ (reverse);

GAPDH: 5′-TGCACCACCAACTGCTTAGC-3′ (forward);

5′-GGCATGGACTGTGGTCATGAG-3′ (reverse).

### 4.16. Auto Docking

This study employs a grid box to investigate the interaction between the AMPK protein and a ligand. The molecular docking analysis between the protein and ligand was performed using AutoDock 4.2 software based on the Lamarckian Genetic Algorithm (LGA). The visualization was performed using PyMol 2.3.2 software, and the most optimal docking position was saved as an image file.

### 4.17. Cellular Thermal Shift Assay (CETSA)

The cells were collected using trypsin and then suspended in a pre-cooled PBS solution with 1% protease inhibitor. The samples were separated into five PCR tubes and exposed to a specific temperature gradient using a PCR amplifier. This involved heating them for 5 min, incubating them at ambient temperature for 5 min, and then freezing them in liquid nitrogen. The cells were subsequently disrupted by liquid nitrogen, which subjected them to two freeze/thaw cycles. The lysate was centrifuged at 20,000× *g* for 20 min to extract the sample, which was then examined by Western blotting.

### 4.18. Drug Affinity Responsive Target Stability (DARTS)

OC cells were collected and lysed using Triton X-100, followed by centrifugation at 15,000 rpm for 15 min. The lysate was divided into five aliquots and treated with DMSO and different concentrations of FX (5, 10, and 20 μM) at room temperature for 1 h. After incubation, proteases (1:1000) were added and incubated at room temperature for 30 min. Finally, 5× loading buffer was added to denature the protein, and the expression of AMPK was detected by Western blotting.

### 4.19. In Vivo Tumor Xenograft Model

Subcutaneously transplanted tumors were initiated by injecting 2 × 10^6^ A2780 cells into the right armpit of 6-week-old female BALB/c nude mice. After the tumors grew to an average volume of 100 mm^3^, the mice were randomly split into two groups. One group received 100 μL of a regular solvent, while the other group was given 100 mg/kg of FX every two days through the stomach. The mice’s body weight, tumor diameter, and tumor volume were observed at intervals of 2 days. The tumor volume was determined using the following formula: tumor volume = length multiplied by the square of the width, divided by 2. After 4 weeks, the animals were euthanized, and the subcutaneous tumors were excised for subsequent analyses.

### 4.20. Immunohistochemistry (IHC) Assay

Immunohistochemistry (IHC) staining was conducted using anti-GPX4 and anti-SLC7A11 antibodies.

### 4.21. Statistical Analysis

The statistical analysis was conducted using the GraphPad Prism software (version 8.0). The data were reported as the mean value ± the standard error of the mean (SEM). The statistical analysis included one-way ANOVA and the *t*-test. Statistical significance was determined when the *p*-value was less than 0.05.

## 5. Conclusions

In conclusion, FX activates apoptosis, pyroptosis, and necroptosis, thereby triggering PANoptosis. Meanwhile, our investigation revealed that FX reduced the activity of GPX4 and SLC7A11, leading to mitochondrial dysfunction and excessive ROS, Fe^2+^, and MDA production, ultimately resulting in ferroptosis. Furthermore, we investigated the mechanism by which FX induces cell death in OC cells through in vivo and in vitro experiments and found that the mechanism of this action was associated with the AMPK/Nrf2/HMOX1 pathway activation of ferroptosis. These findings provide a novel understanding of the processes that cause FX’s inhibitory effects on the growth of OC.

However, this study has several limitations. First, the crosstalk between PANoptosis and ferroptosis pathways, though hypothesized, requires further validation using pathway-specific genetic knockout models. Second, our in vivo experiments utilized xenograft models, which may not fully recapitulate the complexity of the human ovarian tumor microenvironment. Future studies should prioritize the following: (1) elucidating the temporal and spatial interplay between PANoptosis and ferroptosis using single-cell sequencing or live-cell imaging; (2) investigating the synergistic effects of FX with PARP inhibitors and other molecularly targeted therapies to evaluate its potential in combination therapies. Additionally, investigating FX’s efficacy in resistant OC models would clarify its utility in overcoming drug tolerance. These efforts will advance FX’s translation as a multi-targeted therapeutic agent for ovarian cancer.

## Data Availability

All data are available in the article.

## Supplementary Materials

The following supporting information can be downloaded at: https://www.mdpi.com/article/10.3390/md23030123/s1, Figure S1: Cytotoxicity profile of fucoxanthin (FX) in normal cell; Figure S2: PPI network related to FX was constructed; Figure S3: Identification of differentially expressed proteins (DEPs); Figure S4: Relative protein expression of HMOX1, p-AMPK, and Nrf2 after FX treatment in A2780 cells and SKOV3 cells; Figure S5: CETSA showed AMPK target engagement in ovarian cancer cells; Figure S6: AMPK inhibition modulates FX-induced cytotoxicity in A2780 cells (A) and SKOV3 cells (B); Figure S7: Relative protein expression of HMOX1, p-AMPK, and Nrf2 after FX and Compound C treatment in A2780 cells and SKOV3 cells; Figure S8: Relative protein expression of HMOX1 and Nrf2 after FX and ML385 treatment in A2780 cells and SKOV3 cells; Figure S9: Relative protein expression of HMOX1, p-AMPK, and Nrf2 after FX treatment in tumor tissue.

## Abbreviations

CC	Compound C
DEPs	Differently expressed proteins
Fer-1	Ferrostatin-1
FX	Fucoxanthin
GAPDH	Glyceraldehyde 3-phosphate dehydrogenase
IC_50_	The half inhibitory concentration
GPX4	Glutathione peroxidase 4
HO-1-i-1	Heme oxygenase-1-IN-1
LDH	Lactate dehydrogenase
PCD	Programmed cell death
PPI	Protein–protein interaction
SLC7A11	Solute carrier family 7 member 11
IHC	Immunohistochemistry
MDA	Malondialdehyde
MMP	Mitochondrial membrane potential
MTT	Methylthiazolyldiphenyl-tetrazolium bromide
Nec-1	Necrostatin-1
OC	Ovarian cancer
ROS	Reactive oxygen species
TEM	Transmission electron microscopy
