# Supplementary material for: Fucoxanthin from Laminaria japonica Targeting PANoptosis and Ferroptosis Pathways: Insights into Its Therapeutic Potential Against Ovarian Cancer
Source: Mar Drugs. 2025 Mar 12;23(3):123. doi: 10.3390/md23030123 (PMC11943678; doi:10.3390/md23030123)
Supplement: Supplementary file 1 [file marinedrugs-23-00123-s001.zip › marinedrugs-3494372-supplementary.pdf]

Supporting Information

Fucoxanthin from *Laminaria japonica* Targeting PANoptosis and Ferroptosis Pathways: Insights into Its Therapeutic Potential Against Ovarian Cancer

Yaze Wang[1], Yiru Mao[1], Hui Liu[1,2], Yi Huang[,3], and Rong Xu[*,1,2]**

1 Department of Pharmacology, School of Basic Medicine, Tongji Medical College, Huazhong University of Science and Technology, Wuhan, 430030, China; yaze8263@163.com (Y.W.); u202113286@hust.edu.cn (Y.M.); liuh@hust.edu.cn (H.L.)

2 The Key Laboratory for Drug Target Researches and Pharmacodynamic Evaluation of Hubei Province, Wuhan, 430030, China; liuh@hust.edu.cn (H.L.); rongxu@hust.edu.cn (R.X.)

3 Biomedical Analysis Center, Army Medical University, Chongqing, 400038, China; huangyi@tmmu.edu.cn

* Correspondence: huangyi@tmmu.edu.cn (Y. H.), rongxu@hust.edu.cn (R. X.).

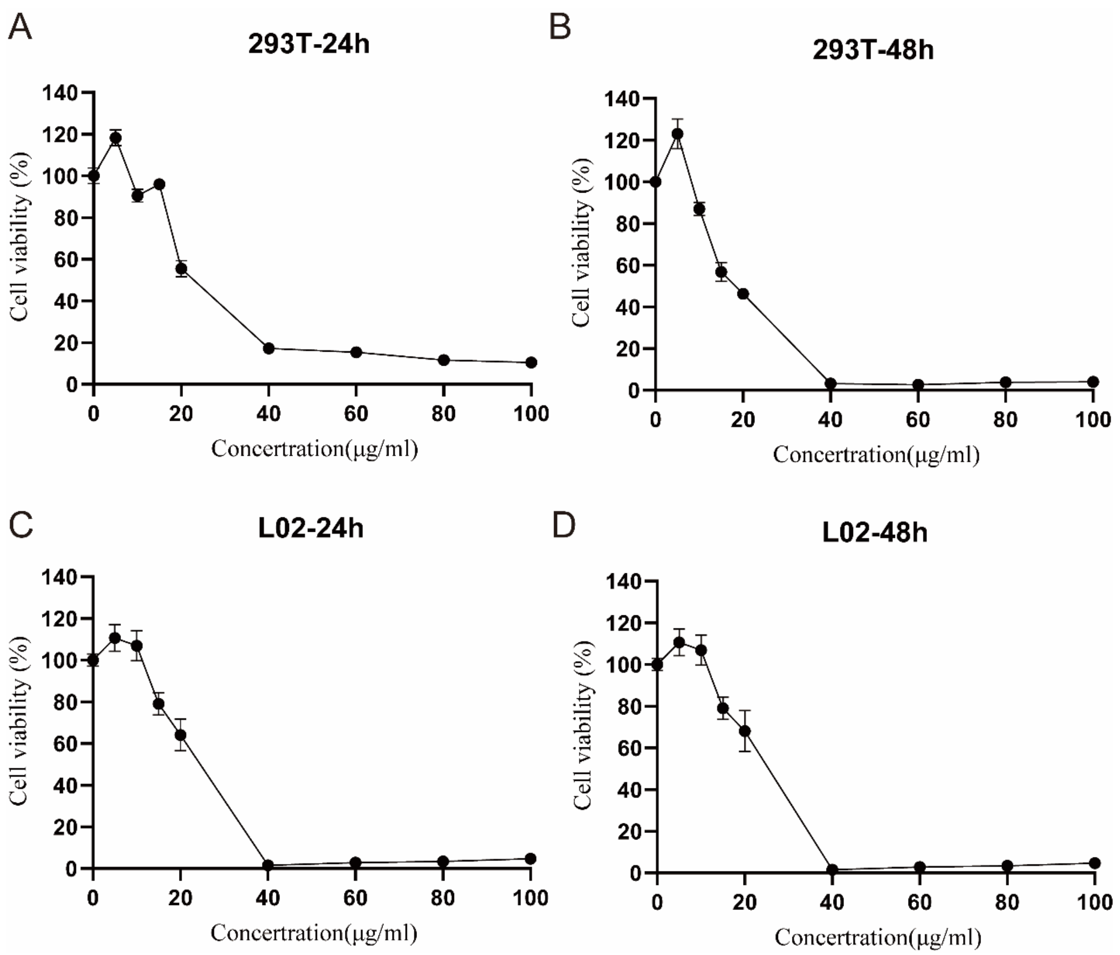

Figures S1. Cytotoxicity profile of fucoxanthin (FX) in normal cell. Cell viability was assessed in 293T (A and B), L02 (C and D) after 24 h and 48 h treatment with FX (0–100 μM).

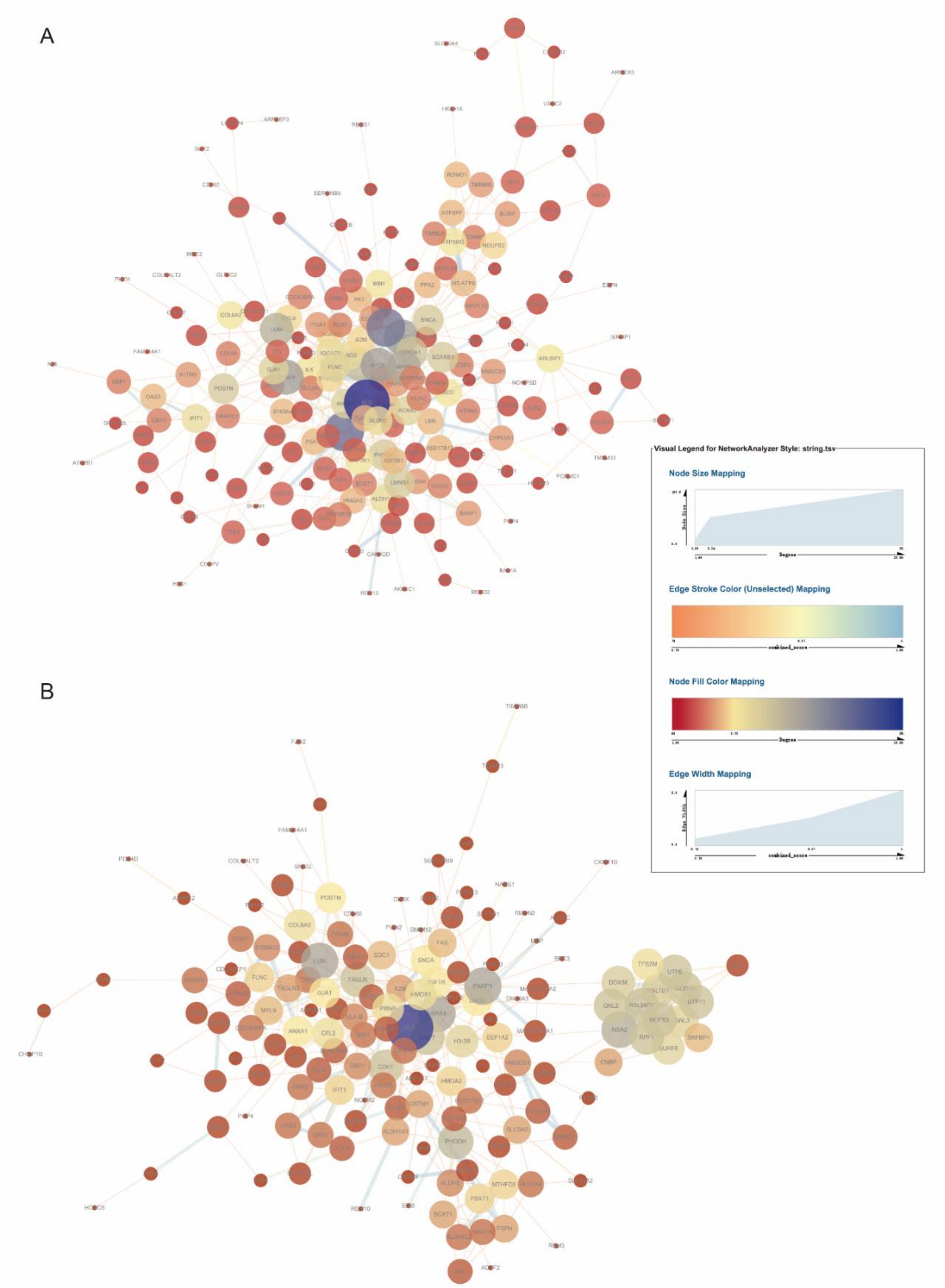

Figures S2. The PPI network related to FX was constructed. (A) The PPI network of 10 μM FX treatment group. (B) The PPI network of 20 μM FX treatment group.

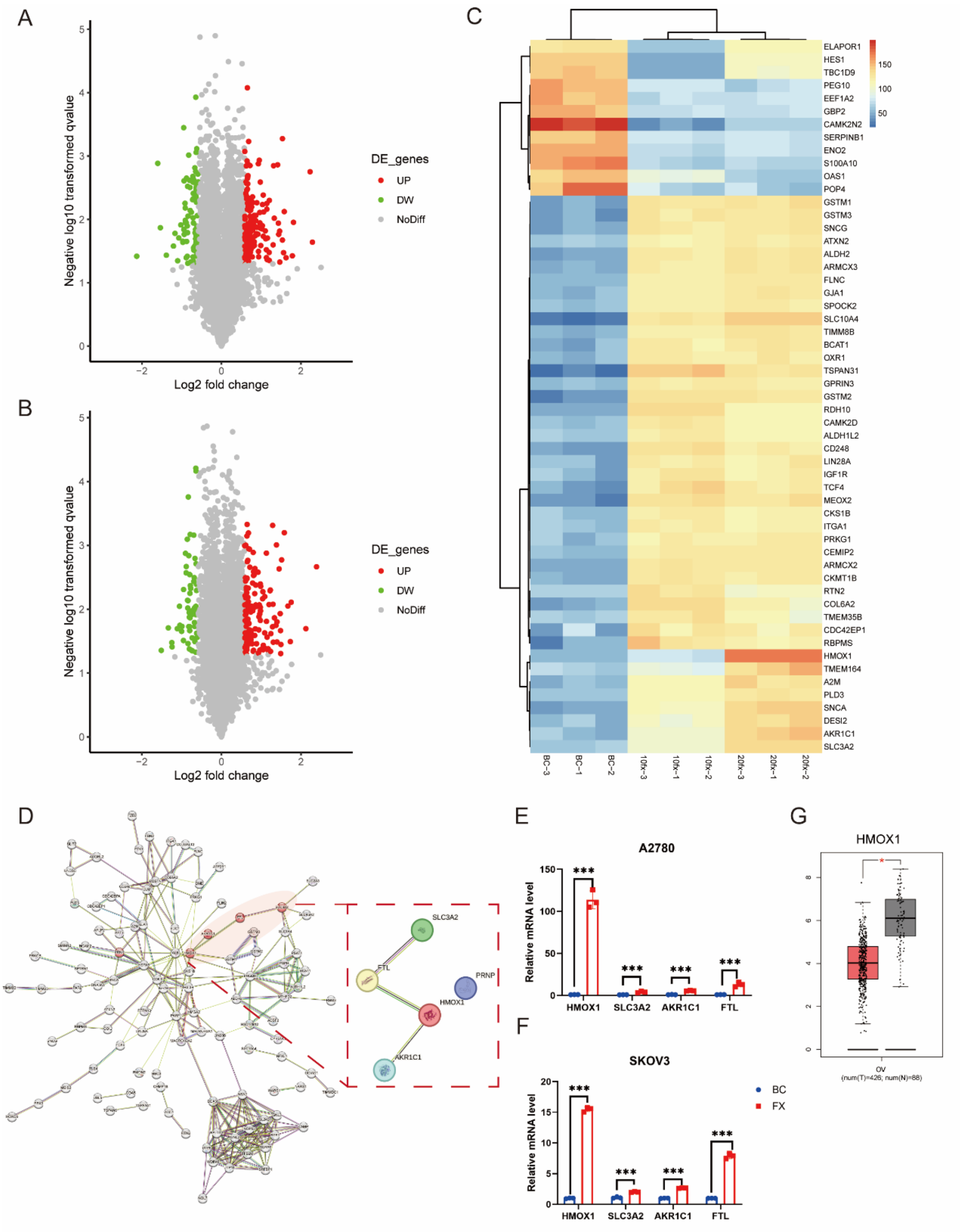

Figures S3. Identification of Differentially expressed proteins (DEPs). (A and B) The Volcano plot of DEPs of 10 μM and 20 μM FX treatment group was identified by DIA analysis using the thresholds of fold change >1.5 and P value<0.05. (C) Heatmap of the expression ratio of DEPs among the control, 10 μM FX treatment group and 20 μM FX treatment groups. The color bar indicates the expression ratio between up-regulated (red) and down-regulated (blue) DEPs. (D) The ferroptosis-related proteins were screened based on the PPI network. (E, F) The mRNA levels of HMOX1, AKR1C1, SLC3A2, and FTL in A2780 and SKOV3 cells. (G) Analysis of HMOX1 expression in ovarian cancer and normal tissues from the GEPIA database. * $p < 0.05$, ** $p < 0.01$, *** $p < 0.001$ vs. control group.

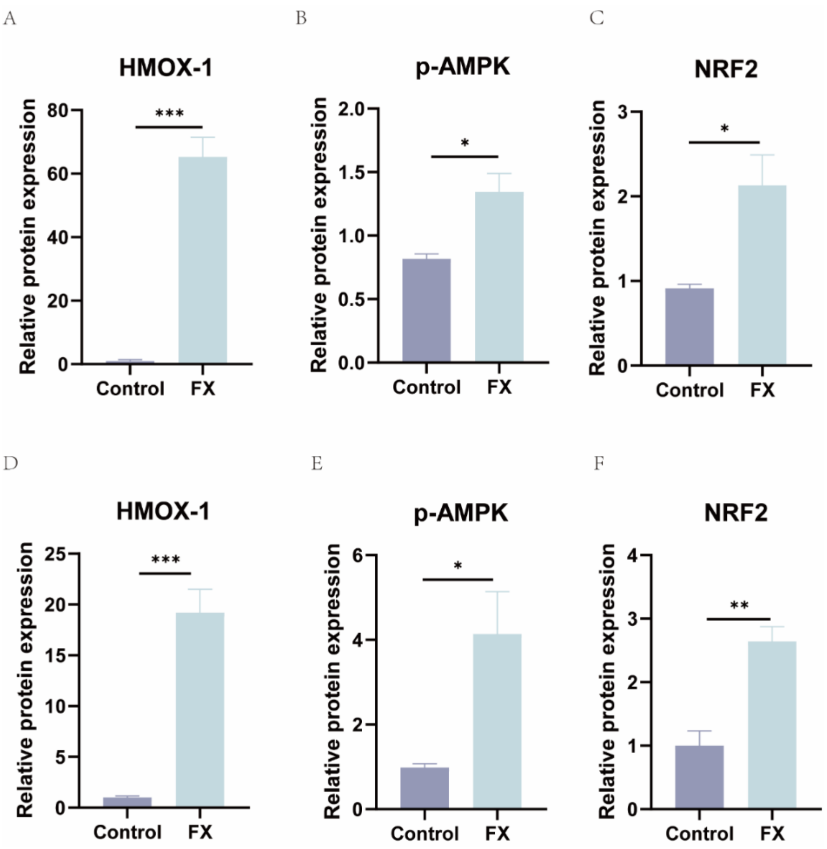

Figures S4. Relative protein expression of HMOX1, p-AMPK, and Nrf2 after FX treated in A2780 cells (A, B, C) and SKOV3 cells (D, E, F).

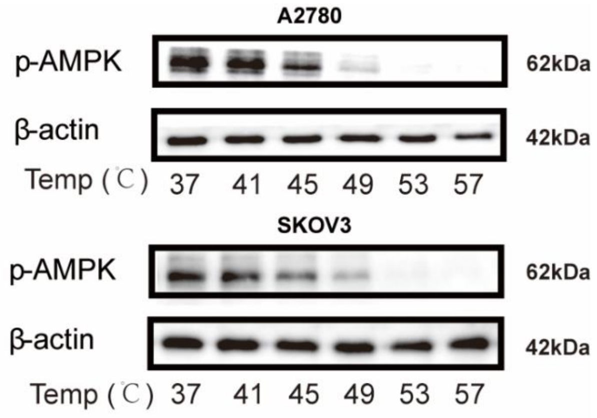

Figures S5. CETSA showed AMPK target engagement in ovarian cancer cells.

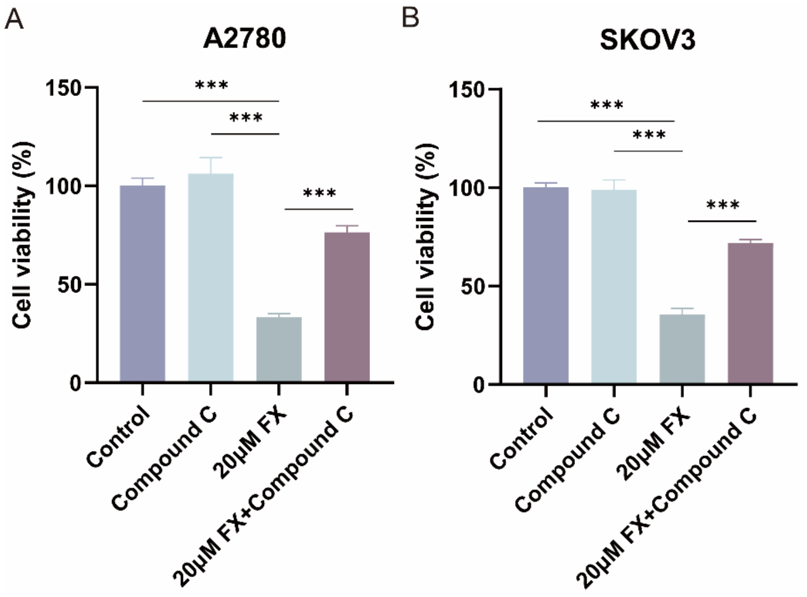

Figures S6. AMPK inhibition modulates FX-induced cytotoxicity in A2780 cells (A) and SKOV3 cells (B).

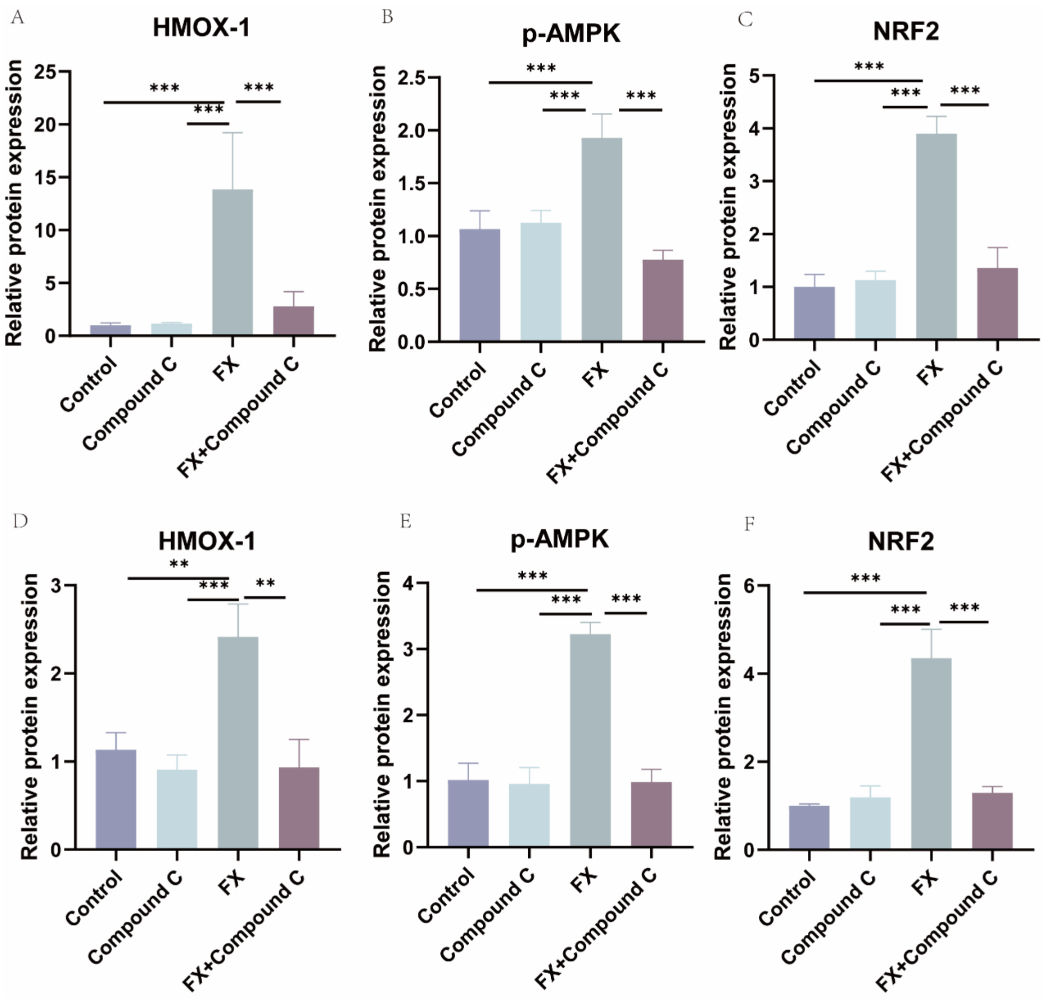

Figures S7. Relative protein expression of HMOX1, p-AMPK, and Nrf2 after FX and compound C treatment in A2780 cells (A, B, C) and SKOV3 cells (D, E, F).

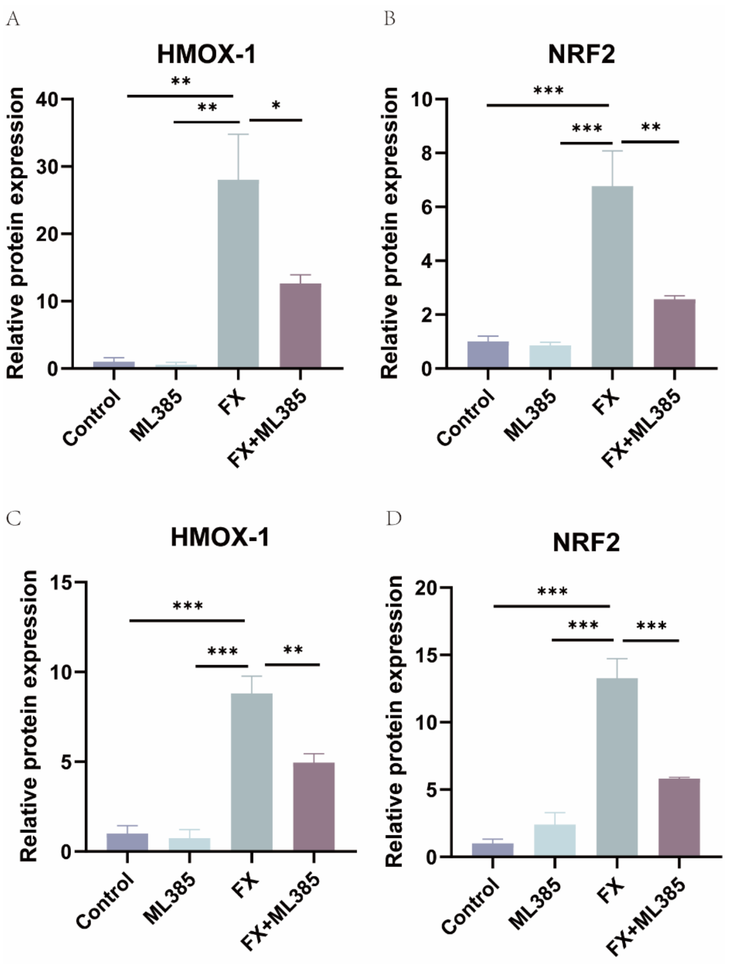

Figures S8. Relative protein expression of HMOX1 and Nrf2 after FX and ML385 treatment in A2780 cells (A, B) and SKOV3 cells (C, D).

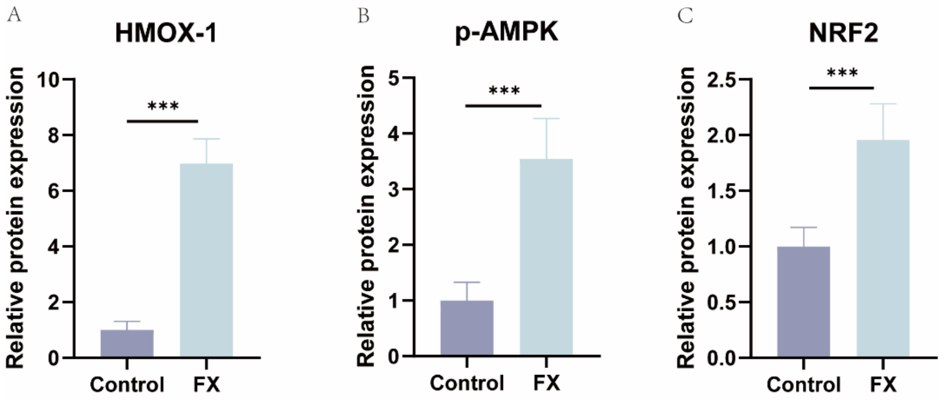

Figures S9. Relative protein expression of HMOX1, p-AMPK, and Nrf2 after FX treatment in tumor tissue.
